# Combinatorial Anatomic and Functional Neural Tract Mapping for Stereotactic Radiosurgery Planning

**DOI:** 10.7759/cureus.6161

**Published:** 2019-11-14

**Authors:** Robert A Scranton, Kuan Yin Hsiao, Saeed S Sadrameli, Hui-Chuan Wang, Yvonne Thong, Patricia Garcia Luzardo, Steve H Fung, Ramiro Pino, Andrew M Farach, E. Brian Butler, Bin Teh, Robert C Rostomily

**Affiliations:** 1 Neurosurgery, Goodman Campbell Brain and Spine, Indianapolis, USA; 2 Radiation Oncology, Houston Methodist Hospital, Houston, USA; 3 Neurosurgery, Houston Methodist Neurological Institute, Houston, USA; 4 Radiation Oncology, University of Texas, Dallas, USA; 5 Radiology, Houston Methodist Hospital, Houston, USA; 6 Neurosurgery, Houston Methodist Hospital, Houston, USA

**Keywords:** diffusing tensor imaging, stereotactic radiosurgery, srs, brain metastases

## Abstract

Introduction

Stereotactic radiosurgery (SRS) is effective and safe for the treatment of the vast majority of brain metastases (BMs). SRS is increasingly used for the simultaneous treatment of multiple lesions, retreatment of recurrence, or subsequent treatment of new lesions. Although radiation injury is relatively uncommon, with the increased utilization of SRS, it is imperative to develop approaches to assess and mitigate radiation-induced neurologic toxicity. Multiple factors influence the development of radiation injury, including patient age, genomic variations, prior treatment, dose and volume treated, and anatomic location. Functional neural structure proximity to SRS targets is a critical factor in developing a systematic integrated risk assessment for SRS patients.

Methods

We developed an approach for risk assessment based on the combinatorial application of i) the anatomic localization of target lesions using a reference neuroanatomical/functional imaging atlas merged with patient-specific imaging and ii) validation with functional MRI (fMRI) and diffusion tensor imaging MRI (DTI-MRI) to identify neural tracts.

Results

In the case of a thalamic/midbrain junction breast carcinoma metastasis, the reference image analysis revealed proximity to the corticospinal tract (CST), which was validated by functional DTI-MRI. Dose-volume exposure of the CST could be estimated and considered in the development of a final treatment plan.

Conclusion

Merging pretreatment MR imaging with neuroanatomical/functional reference MRIs and subsequent validation with fMRI or DTI-MRI may prove to be a valuable approach to screen for neural risks in individual SRS patients. Incorporating this approach in larger studies could further our understanding of dose tolerances in a broad range of neural structures.

## Introduction

Stereotactic radiosurgery (SRS) has proven to be a safe and effective treatment modality for many brain lesions and has become the most common treatment option for brain metastases. The challenge of SRS is to deliver a therapeutic dose to the intended target while minimizing toxicity to surrounding structures. Factors that influence the toxicity risk, and which must be accounted for in generating a treatment plan, include fractionation, margin dose, dose volume, patient age, and intrinsic radiation tolerance for specific neural structures [1-2]. Therefore, techniques that can readily provide anatomic risk assessments of peri-lesional neural structures may be of great usefulness in treatment planning and avoidance of neurologic morbidity from SRS.

The neurological structures most commonly evaluated for radiation toxicity include the optic apparatus, cochlea (for internal auditory canal lesions), and the brain stem. These structures can be readily identified on routine magnetic resonance (MR) imaging and segmented for dose risk assessment, but functional neural tracts, such as the corticospinal tracts (CSTs), are less readily identifiable with standard MR imaging. Functional MR imaging (fMRI) and diffusion tensor imaging (DTI-MRI) have emerged as tools to localize speech/language and motor cortex and neural tracts supporting language (i.e. arcuate fasciculus) and motor function (CSTs) [3-4]. To complement fMRI and DTI-MRI for anatomic neural risk assessment, our institution has developed a three-dimensional stereological atlas of neural structures, Plato’s computer augmented virtual environment (CAVE), which can be readily merged with patient-specific imaging [5]. With this system, neural structures in proximity to intended SRS targets can be visualized to provide a first approximation of potential functional risk, which can be further validated if necessary with advanced fMRI and DTI-MRI studies for SRS planning. 

Here, we present the combined use of Plato’s CAVE and DTI-MRI imaging to identify the anatomy of the CST as an adjunct for the linear accelerator (LINAC)-based SRS treatment planning of solitary brain metastasis in the thalamus/mid-brain junction. The case demonstrated the feasibility of integrating Plato’s CAVE and DTI-MRI, as well as the dose-volume histogram (DVH) of the adjacent fiber pathway, as a potential tool to mitigate neural tract toxicity in SRS.

## Materials and methods

Clinical history

**Figure 1:**
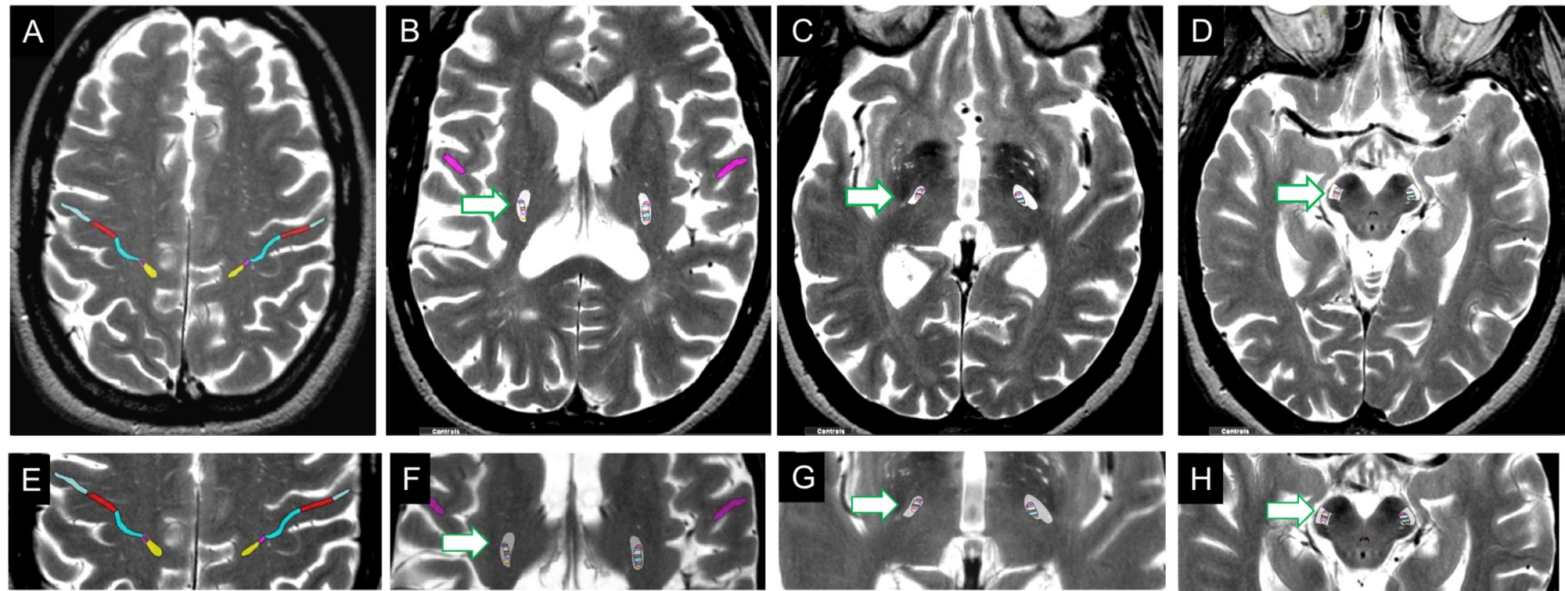
Functional-anatomical localization using Anatom-e The primary motor cortex (A, F) and corticospinal tracts (B-D, F-H) are shown on a reference T2-weighted MRI. The somatotopic organization of each is indicated by different colors (yellow-leg; purple-trunk or shoulder; light blue-shoulder/arm; red/lighter blue- forehead and face). The CTS is outlined in white (indicated by the white arrow with a green outline) at the levels of the centrum semiovale (B,F), basal ganglia (C,G), and midbrain peduncle (D,H). CST: corticospinal tract

The patient was an 89-year-old female, with T2N2Mx invasive ductal carcinoma of the right breast, who was treated with modified radical mastectomy in May 2015 and postoperative chemotherapy. She presented with gait imbalance and a new chronic headache in January 2017. At this time, her neurologic examination was unremarkable except for mild symmetric lower extremity weakness but was able to ambulate unaided. A brain MRI in March 2017 demonstrated a 1.7 cm x 1.7 cm x 1.7 cm (2.6 ml total volume) ring-enhancing mass in the left ventral thalamus-midbrain junction with associated edema and mass effect consistent with metastatic breast cancer (Figure 1). On fluorodeoxyglucose positron emission tomography (FDG PET) imaging, increased focal uptake in the left thalamus was observed with no evidence of another systemic disease.

Combined neuro-anatomic and functional imaging (DTI-MRI)

**Figure 2:**
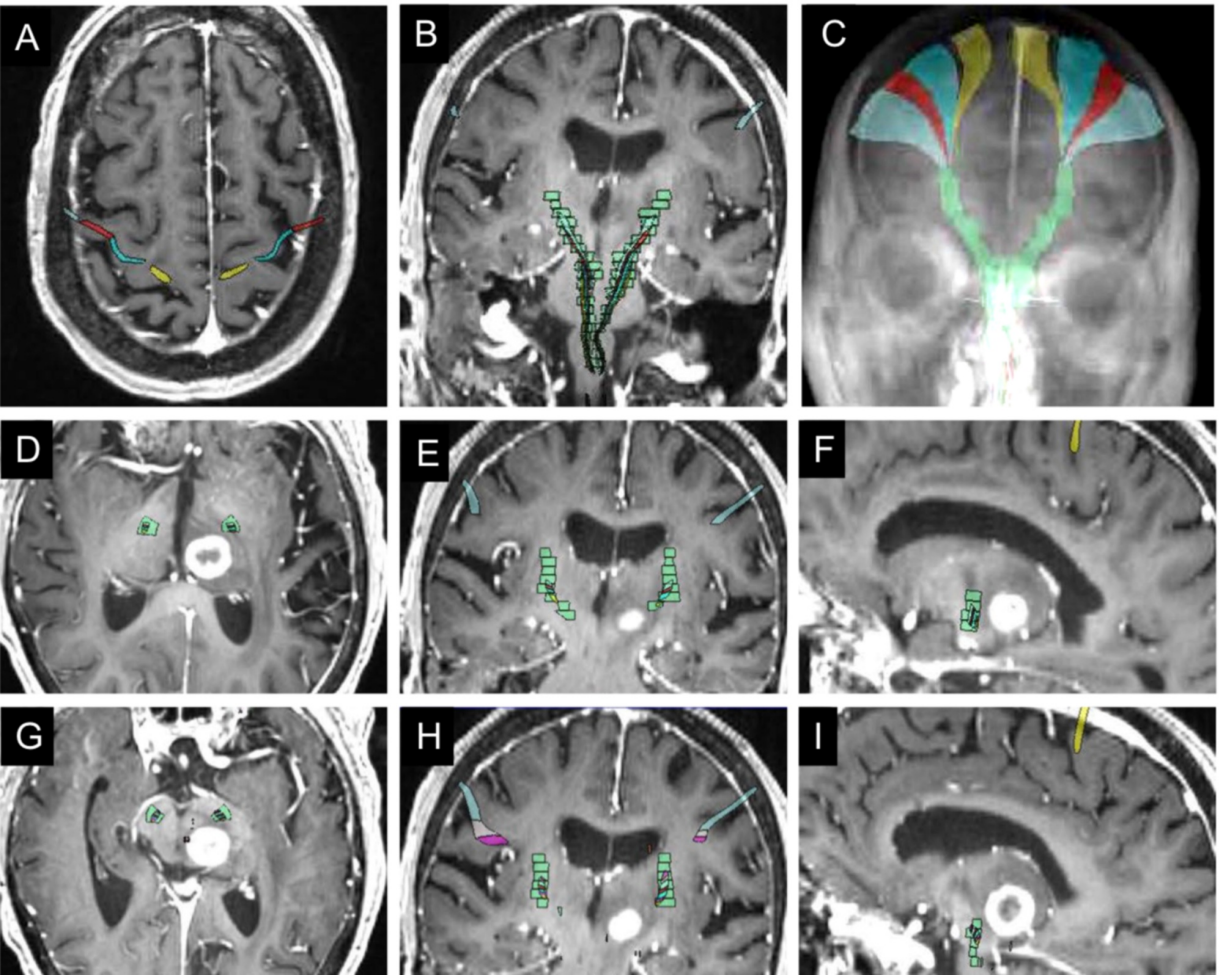
Proximity of target metastasis to corticospinal tracts (CST) using co-registration of diagnostic MRI and Anatom-e atlas (A-C) Patient diagnostic MRI merged to Anatom-e defined motor cortex (A) and CST (B) with 3D somatotopic rendering of motor fibers coalescing into the CST (green) (C). (D-I) Co-localization of target lesion with CST (green) in axial (D, G), coronal (E,F) and sagittal (F,I) planes.

To define potential anatomic risks, we merged the pre-treatment gadolinium-enhanced MR image with a reference MRI on which functional elements can be segmented using a commercially available functional/anatomically annotated neuro-radiologic atlas (Anatom-e; http://anatom-e.com/). A representation of the corticospinal tract and somatotopic fiber tracts segmented in the reference MRI is shown in Figure 2.

**Figure 3:**
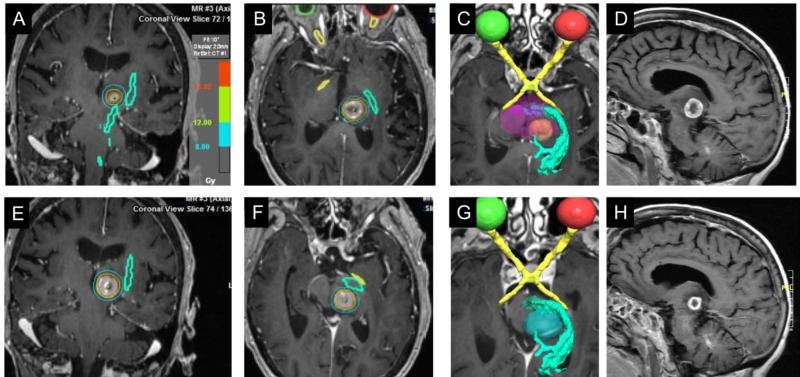
SRS treatment planning using DTI-MRI images to localize corticospinal tracts For SRS treatment planning, an MRI with DTI was used to establish the proximity of the target lesion to the CST and calculate dose-volume histograms for the CST. Coronal (A,E) and axial (B,F) images from the SRS treatment plan showing MRI-DTI defined CST (green), gross tumor volume (GTV; orange), and isodose lines (blue - 8Gy; green - 12 Gy; red - 14Gy). (C,G) Axial 3D renderings of CTS (bright green) relative to the GTV (C) and 8 Gy isodose volume (G). The optic pathway is outlined (yellow; C,D) as well as the brainstem (purple; C). Pretreatment (D) and one-month post-treatment (H) MRI with gadolinium show a marked reduction in tumor volume. DTI: diffusion tensor imaging; CST: corticospinal tract

After manual merging to the reference MRI, the location of CSTs was visualized in the pre-treatment MRI (Figure 3). This analysis demonstrated that the lesion was within 1-2 mm of the CST based on the reference functional neuroanatomic atlas. Given the proximity of the lesion to the CST, a DTI-MRI was then obtained to assist in treatment planning.

DTI-MRI was then performed to provide functional validation of the CTS localization. Brain MRI was acquired using an eight-channel head coil on a 3.0T clinical MRI scanner (Discovery MR750, GE Healthcare, Waukesha, Wisconsin, USA). Structural images included 3D T1-weighted inversion recovery-prepared fast spoiled gradient echo (BRAVO) with parameters TR 8.2ms, TE 3.2 ms, TI 450ms, flip angle 12°, field of view (FOV) 24 cm, acquisition matrix 256x256, and slice thickness 1.5 mm after intravenous administration of 0.1 mmol/kg gadobutrol (Gadavist, Bayer Healthcare, Whippany, New Jersey, USA). DTI was obtained with parameters TR 14000ms, TE 84.4ms, b-value 0,1000 s/mm2, 15 diffusion gradient directions, FOV 24 cm, acquisition matrix 128x128, and slice thickness 2.6 mm. Fiber tractography was performed by a neuroradiologist using Diffusion Toolkit and TrackVis (MGH/HST Athinoula A. Martinos Center for Biomedical Imaging, Charlestown, Massachusetts, USA) with second-order Runge-Kutta propagation algorithm, 35° angle threshold, minimum fractional anisotropy threshold automated by software. Co-registered white matter tracts were then exported as digital imaging and communications in medicine (DICOM) objects to picture archiving and communication system (PACS) and integrated into the treatment planning system to visualize the adjacent fiber tracts with special attention paid to the left corticospinal tract (CST). The localization of the CST relative to the brain metastasis was very similar when comparing the reference MRI study to the DTI-MRI. In both, portions of the CST were localized within 1-2 mm of the CST.

## Results

The BrainLab (Munich, Germany) radiosurgery mask and BrainLab head and neck localizer were applied to immobilize the patient during simulation and treatment. A non-contrast CT scan with 1.5 mm axial slices was acquired. High-resolution brain MRI with stereotactic radiosurgery protocol and CST DTI tractography was fused for targeting and planning as described above. Target and critical structure delineation was performed on the postcontrast brain volume (BRAVO) images and fused with the CT scan. The relationship between the gross target volume (GTV) of the tumor and the CST was identified by DTI. The neurosurgeon reviewed and finalized the defined target and structures to be preserved. The SRS plan was generated with a single isocenter by seven non-coplanar dynamic conformal arcs (iPlan®RT by BrainLab, Heimstetten, Germany). Margin doses of 16 Gy are reported to provide safe and effective control of brainstem metastases but since toxicity is increased in the elderly and with treatment volumes >2 cc, we chose to prescribe 14 Gy to the 90% gross tumor volume (GTV) of 3.109 cc [6]. The isodose line and dose-volume histograms were generated to evaluate target coverage and critical structure doses as well as their anatomic relationships with the CST. The maximum dose to the CST was 12.98 Gy with 0.008 cc receiving 12 Gy and 0.064 cc receiving 8 Gy. The maximum brain stem dose was 14.39 Gy with 0.592 cc receiving 10 Gy. SRS was delivered without complication by BrainLab Novalis SystemTM (Heimstetten, Germany) using ExacTrac (BrainLab, Heimstetten, Germany) and image guidance.

At the one-month follow-up, the patient was doing well with no new neurological symptoms and resolution of the headache. Follow-up brain MRI showed a decrease in the size of the treated solitary brain metastasis. There was no edema or mass effect and no new lesions were detected. Due to the progression of her systemic disease, no further follow-up imaging was obtained.

## Discussion

The goal of SRS in patients with brain metastasis (BM) is to enhance the quality of life and survival by maximizing tumor control and either eliminating or minimizing the potential impacts of radiation injury. This goal must be realized in the context of enhanced survival and primary disease control and increasing BM incidence and the consequently increased utilization of SRS [7]. Therefore, it is of importance to develop systematic approaches to mitigate radiation injury, increase our understanding of factors contributing to neural toxicity, and, eventually, generate more predictive models of risk assessment as the clinical landscape of metastatic brain cancer evolves. While important observations have been made regarding dose tolerance and risk profiles for some structures (i.e. optic apparatus, brain stem, and cochlea), less is known about other functional neural structures such as the corticospinal tract (CST) [8-12]. Here, we presented a case study of SRS treatment of a thalamic/midbrain junction BM adjacent to the CST to highlight a proposed imaging-based algorithm for neural risk assessment in SRS patients, which may be useful for treatment planning and prospective clinical studies.

The proposed risk assessment algorithm is a two-step process. First, clinical SRS treatment planning MR images are imported into a reference functional neuro-anatomic atlas (Anatom-e) to screen for potential at-risk structures. In the present case, the CST was readily identified as a potential at-risk structure for SRS. Based on this analysis, patients deemed to be at high risk for potential functional radiation-related toxicity undergo fMRI and/or tractography DTI-MRIs as indicated. Merged functional and treatment planning MR imaging then provide patient-specific imaging of functional anatomical relationships to target lesions. In our case, the proximity of the CST to the target lesion was confirmed by merging the treatment planning MRI to the DTI-MRI. The availability of imaging-based functional anatomic resources and standardized functional and DTI MR imaging at most centers supports the prospective application of this paradigm to refine our understanding of SRS-mediated functional toxicity. This approach is expected to allow clinicians to better mitigate neural toxicity by modifying treatment planning according to current guidelines for dose-volume tolerances of functional structures.

In the current report, we mapped the CSTs prior to SRS treatment planning. Previous studies have proposed the use of CST DTI imaging to assist in SRS treatment planning [13-17]. For arteriovenous malformations (AVMs) treated with SRS, motor deficits correlate with higher doses and volumes treated as well as location within the CST (corona radiata more resistant than the internal capsule) [17]. Koga et al. (2012) reported that CST doses <20 Gy significantly reduced the incidence of motor complications from 17.9% to 4.2% [15]. In our case, CST doses were well below these levels (maximum dose of 12.98 Gy) but potential biological differences between AVM and BMs and the short follow-up preclude the assessment of CST tolerance or complication avoidance. To refine our understanding of functional risks from BM SRS treatment, it would be of great potential value to prospectively incorporate dose-volume data for functional structures in larger radiosurgery databases such as the NeuroPoint Alliance [18]. This large-scale analysis could inform guidelines for BM treatment based on SRS delivery type (LINAC vs Gamma Knife), proximity to a broad range of functional structures (cortical as well as white matter tracts) and account for the effects of the primary cancer (histology, stage, molecular profile), MR imaging (edema, necrosis, cysts, metabolic signatures), and clinical factors (age, sex, prior and concurrent therapies, performance status) among others.

For lesions situated near more complex functional structures such as the skull base, it may also be important to extend the pre-treatment risk assessment using more robust imaging and virtual reality approaches. For instance, integrating multiple imaging modalities in space and time along with proposed treatment plans with an image-guided visualization system, such as Plato’s CAVE, can render an anatomically and functionally enriched 3D virtual reality of a BM, its surrounding structures, and their potential risk profiles [5]. This application of a cross-reality system (CRS) can make full use of the strength of each imaging modality (CT, MRI, and PET) by fusing all of the images available for an individual patient [19]. This technology is also helpful in combining molecular images and gene expression cluster analysis with the clinical images that may inform future patient-specific combinatorial SRS-systemic treatment paradigms in the future [20].

As the treatment of cancer outside of the central nervous system (CNS) improves, the incidence of BMs is expected to increase along with the use of SRS. Current evidence supports the use of SRS as opposed to whole-brain radiotherapy to preserve cognitive function and enhance the quality of life in BM patients [21-22]. However, radionecrosis (RN) occurs in a substantial number of patients after SRS for BMs with reported rates of ~5%-40% [23-25]. Some studies reported that the risk of radiation-induced deficits after SRS was 12%-19% for lesions in the thalamus, basal ganglia, and brain stem, which was much higher than that for lesions in the motor cortex [26-28]. In one case series of BM patients treated with SRS and concomitant systemic therapies, 8% developed RN of which 54% were symptomatic [23]. Therefore, it is important to further refine risk stratification for symptomatic neural toxicity from RN in order to optimize oncologic and neural functional outcomes in the ever-increasing population of patients with BMs. We propose that the prospective application of the algorithm for risk assessment presented here can be a valuable tool to achieve this goal.

## Conclusions

Merging pretreatment MR imaging with neuroanatomical/functional reference MRIs and subsequent validation with fMRI or DTI-MRI may prove to be a valuable approach to screen for neural risks in individual SRS patients. Incorporating this approach in larger studies could further our understanding of dose tolerances in a broad range of neural structures.

## References

[1] Kirkpatrick JP, Soltys SG, Lo SS, Beal K, Shrieve DC, Brown PD (2017). The radiosurgery fractionation quandary: single fraction or hypofractionation?. Neuro Oncol.

[2] Patel A, Dong T, Ansari S (2018). Toxicity of radiosurgery for brainstem metastases. World Neurosurg.

[3] Viallon M, Cuvinciuc V, Delattre B (2015). State-of-the-art MRI techniques in neuroradiology: principles, pitfalls, and clinical applications. Neuroradiol.

[4] Filippi M, Agosta F (2016). Diffusion tensor imaging and functional MRI. Handb Clin Neurol.

[5] Butler EB, Sovelius P, Huynh N (2013). Plato’s CAVE a multidimensional, image-guided radiation therapy cross reality platform with advanced surgical planning, simulation, and visualization techniques using (native) DICOM patient study studies. Computational Surgery and Dual Training.

[6] Trifiletti DM, Lee CC, Kano H (2016). Stereotactic radiosurgery for brainstem metastases: an international cooperative study to define response and toxicity. Int J Radiat Oncol Biol Phys.

[7] Fidler IJ (2011). The role of the organ microenvironment in brain metastasis. Semin Cancer Bio.

[8] Doroslovacki P, Tamhankar MA, Liu GT, Shindler KS, Ying GS, Alonso-Basanta M (2018). Factors associated with occurrence of radiation-induced optic neuropathy at "safe" radiation dosage. Semin Ophthalmol.

[9] Kirkpatrick JP, Marks LB, Mayo CS, Lawrence YR, Bhandare N, Ryu S (2011). Estimating normal tissue toxicity in radiosurgery of the CNS: application and limitations of QUANTEC. J Radiosurg SBRT.

[10] Linskey ME (2013). Hearing preservation in vestibular schwannoma stereotactic radiosurgery: what really matters?. J Neurosurg.

[11] Mayo C, Martel MK, Marks LB, Flickinger J, Nam J, Kirkpatrick J (2010). Radiation dose-volume effects of optic nerves and chiasm. Int J Radiat Oncol Biol Phys.

[12] Xue J, Goldman HW, Grimm J, LaCouture T, Chen Y, Hughes L, Yorke E (2012). Dose-volume effects on brainstem dose tolerance in radiosurgery. J Neurosurg.

[13] Aoyama H, Kamada K, Shirato H, Takeuchi F, Kuriki S, Iwasaki Y, Miyasaka K (2003). Visualization of the corticospinal tract pathway using magnetic resonance axonography and magnetoencephalography for stereotactic irradiation planning of arteriovenous malformations. Radiother Oncol.

[14] Koga T, Maruyama K, Igaki H, Tago M, Saito N (2009). The value of image coregistration during stereotactic radiosurgery. Acta Neurochir.

[15] Koga T, Shin M, Maruyama K (2012). Integration of corticospinal tractography reduces motor complications after radiosurgery. Int J Radiat Oncol Biol Phys.

[16] Maruyama K, Kamada K, Shin M (2005). Integration of three-dimensional corticospinal tractography into treatment planning for gamma knife surgery. J Neurosurg.

[17] Maruyama K, Kamada K, Ota T (2008). Tolerance of pyramidal tract to gamma knife radiosurgery based on diffusion-tensor tractography. Int J Radiat Oncol Biol Phys.

[18] Sheehan JP, Kavanagh BD, Asher A, Harbaugh RE (2016). Inception of a national multidisciplinary registry for stereotactic radiosurgery. J Neurosurg.

[19] Lifton J, Laibowitz M, Harry D, Gong N-W, Mittal M, Paradiso J (2009). Metaphor and manifestation: cross reality with ubiquitous sensor/actuator networks. IEEE Pervasive Comput.

[20] Speers C, Tsimelzon A, Sexton K (2009). Identification of novel kinase targets for the treatment of estrogen receptor-negative breast cancer. Clin Cancer Res.

[21] Brown PD, Ballman KV, Cerhan JH (2017). Postoperative stereotactic radiosurgery compared with whole brain radiotherapy for resected metastatic brain disease (NCCTG N107C/CEC·3): a multicentre, randomised, controlled, phase 3 trial. Brain.

[22] Brown PD, Jaeckle K, Ballman KV (2016). Effect of radiosurgery alone vs radiosurgery with whole brain radiation therapy on cognitive function in patients with 1 to 3 brain metastases: a randomized clinical trial. JAMA.

[23] Kim JM, Miller JA, Kotecha R (2017). The risk of radiation necrosis following stereotactic radiosurgery with concurrent systemic therapies. J Neurooncol.

[24] Le Rhun E, Dhermain F, Vogin G, Reyns N, Metellus P (2016). Radionecrosis after stereotactic radiotherapy for brain metastases. Expert Rev Neurother.

[25] Rahmathulla G, Marko NF, Weil RJ (2013). Cerebral radiation necrosis: a review of the pathobiology, diagnosis and management considerations. J Clin Neurosci.

[26] Pollock BE, Gorman DA, Brown PD (2004). Radiosurgery for arteriovenous malformations of the basal ganglia, thalamus, and brainstem. J Neurosurg.

[27] Andrade-Souza YM, Zadeh G, Scora D, Tsao MN, Schwartz ML (2005). Radiosurgery for basal ganglia, internal capsule, and thalamus arteriovenous malformation: clinical outcome. Neurosurgery.

[28] Hadjipanayis CG, Levy EI, Niranjan A, Firlik AD, Kondziolka D, Flickinger JC, Lunsford LD (2001). Stereotactic radiosurgery for motor cortex region arteriovenous malformations. Neurosurgery.

